# Flow-based method for biofilm microbiota enrichment and exploration of metagenomes

**DOI:** 10.1186/s13568-022-01377-y

**Published:** 2022-03-21

**Authors:** Gunhild Hageskal, Tonje Marita Bjerkan Heggeset, Giang-Son Nguyen, Tone Haugen, Malene Jønsson, Conceição Egas, Aurelio Hidalgo, Alexander Wentzel, Anna Sofia Lewin

**Affiliations:** 1 Department of Biotechnology and Nanomedicine, SINTEF Industry, P.O. Box 4760 Torgard, N-7465 Trondheim, Norway; 2 Center for Neuroscience and Cell Biology, University of Coimbra, Coimbra, Portugal; 3 Centro de Biología Molecular “Severo Ochoa” (UAM-CSIC), Madrid, Spain

**Keywords:** Flow-based enrichment, Biofilm, Microbiome, Metagenomes, Industrial waste substrates

## Abstract

Most bacteria live in biofilms in their natural habitat rather than the planktonic cell stage that dominates during traditional laboratory cultivation and enrichment schemes. The present study describes the establishment of a flow-based enrichment method based on multispecies biofilm communities for directing biofilm functionality using an environmental inoculum. By controlling flow conditions and physio-chemical properties, the set-up aims to simulate natural conditions ex situ for biofilm formation. The functionality of the method was demonstrated by enrichment of biofilm microbiomes using consortia from a warm compost pile and industrial waste materials as growth substrate, and further exploring the metagenomes by biotechnological tools. The 16S rRNA gene sequencing results revealed a difference in consortium composition and especially in genus abundance, in flow experiments compared to traditional liquid-shake experiments after enrichment, indicating good biofilm development and increased abundance of biofilm-forming taxa. The shotgun sequence mining demonstrated that different enzymes classes can be targeted by enriching biofilms on different substrates such as oat husk, pine saw dust, and lignin. The flow-based biofilm method is effective in reducing bacterial consortia complexity and in selecting biofilm-forming bacteria, and it is possible to enrich the biofilm community in various directions based on the choice of sample material, environmental conditions, and nutritional preferences, targeting enzymes or enzyme classes of industrial interest.

## Introduction

Functional metagenomics and bioprospecting of environmental samples has been highlighted as an important scientific approach to search for novel biomolecules with biotechnologically valuable properties that may be applicable e.g. in industrial processes, as well as have the potential to mitigate the effects of climate change (Alivisatos et al. 2015; Dubilier et al. 2015; Long et al. 2016). Focus areas such as green energy, circular economy, bioremediation, recycling, waste management, health challenges like antibiotic resistance, and sustainable agriculture would benefit from increased success rates in discovery of applicable enzymes and other products. Microbial metagenomes, especially from extreme environments, comprise enormous genetic reservoirs encoding enzymes with industrial potential (Simon and Daniel 2011). Unfortunately, harvesting these reservoirs is difficult, first of all due to the fact that less than 1% of the microbes can be cultured under standard laboratory conditions (Epstein 2013), but also because applicable gene candidates are rare and hidden in an overwhelming majority of irrelevant genetic material. Microbial enrichment approaches may help reduce the complexity and diversity of the targeted microbiomes, maintaining the most relevant genomes for finding promising gene and enzyme candidates. The enrichment of specific environmental samples with selected growth substrates will reduce the bacterial richness but hopefully enrich for those bacteria with relevant gene material, e.g. bacteria with the ability to degrade biological waste materials in a hot compost pile. Enrichment strategies are very diverse and can be carried out in the natural habitat (in situ), e.g. in bacterial traps (Gavrish et al. 2008) or diffusion chambers (Nichols et al. 2010), or in laboratory enrichment systems (ex situ), e.g. in shaken liquid medium, in cocultivation systems ensuring presence of critical growth factors (D'Onofrio et al. 2010), or other microcosms systems (Wilhelm et al. 2019).

Most natural bacterial populations are closely associated with environmental surfaces and with each other, and it is acknowledged that multispecies biofilm represents the most natural form of bacterial life (Costerton et al. 1995; Hall-Stoodley et al. 2004). Bacterial biofilm communities in natural environments are subjected to several external physical factors that are difficult to simulate in standard laboratory cultivations, such as the flow of fluids at the community habitat. It has been showed that flow induces biofilm formation in clinical isolates (Weaver et al. 2012). The biofilm consortium can be engineered by shifting environmental factors and nutrient sources, e.g. towards more efficient biodegradation of toxic pollutants (Benedek et al. 2018) and better pesticide degradation (Verhagen et al. 2011). These studies utilises liquid-shake systems, hence examples of enrichment studies using perfusion strategies for biofilm enrichments are scarce.

The present study was designed to establish an ex situ enrichment method based on multispecies biofilm communities for directing biofilm functionality using an environmental inoculum. By applying specific fluidic flow conditions and other physical properties like pH, temperature, and incubation time, as well as nutrient alterations, the goal was to approach natural conditions ex situ, facilitating biofilm formation by microorganisms favoured by certain growth conditions. The main aim was to use this method to enrich biofilm microbiota from a hot compost pile by using different industrial waste materials, and to explore the metagenomes by biotechnological tools.

## Methods and materials

### Method development

The flow-based enrichment method was tested and established using garden soil as inoculum for targeting environmental conditions and for use of a defined carbon source. For ensuring proper biofilm development, different pH conditions (pH 4, pH 5.4, pH 7, and pH 8) were tested, in combination with different incubation temperatures (20 and 30 °C). Substrates like cellulose (Avicell, crystallised) or glucose (Sigma-Aldrich) were used as carbon source, and the biofilm formed was harvested after different incubation times (day 5, day 7, day 10, day 23, and day 38). The output was also compared to more standard shaken-liquid experiments (same inoculum, media and conditions, no flow, 200 rpm), to potentialize the usability of the flow-based strategy. The development of biofilm in the flow cells were also examined in real-time by microscope (EVOS FL Auto Imaging system, LifeTechnologies™, see section “Flow experiments”). A control experiment with the same environmental conditions (pH 8, 30 °C) and the same carbon source (cellulose) in four parallel flow cells was included to demonstrate the reproducibility of the method. The method was then applied to enrich biofilm communities with three different finely ground carbon-rich substrates (oat husk, pine saw dust, and lignin) prepared in Spain (University of Madrid, Dept. of Molecular Biology), and using a microbe inoculum from a Portuguese compost pile (Center for Neuroscience and Cell Biology).

### Samples and substrates

Garden soil from the Trondheim area (Lat. 63° 25′ 10.578'' N, Long. 10° 28′ 18.1128'' E), Norway, was sampled into a sterile 120 ml container and dispersed into four sterile 50 ml tubes, containing soil of the approx. volume of 20 ml in each tube. The tubes were filled with sterile water up to 50 ml, inverted at 125 rpm for one hour, and then centrifuged at 3220×*g* for 10 min. The resulting supernatant was centrifuged once more at 3220×*g* for 10 min to generate a ‘soil extract solution’. A specially designed nutritional solution (per litre; NH_4_Cl 1.15 g, K_2_HPO_4_ 0.3 g, MgSO_4_ ⋅ 7H_2_O 0.015 g, NaH_2_PO_4_ ⋅ 2H_2_O 0.15 g, FeCl_3_ ⋅ 6H_2_O 0.01 g, Carbon source of choice 4 g, and sterile water 1000 g) was added to a final concentration of 10% to the soil extract solution, and the mix was adjusted to the pH of choice (4 and 8). The final inoculum flow medium, consisting of the soil extract and the nutritional solution including selected carbon source, was then injected in the syringes in the flow system.

Compost samples were used for the main demonstration of the method, and the microbe inoculum was extracted from the samples according to the protocol described for garden soil above. The compost samples were collected from a wood compost pile in Portugal (Lat. 40° 22′ 45.840'' N, Long. 8° 36′ 57.24'' W) in September 2019. The pile was composed mainly of pine and eucalyptus scraps, barks, and small branches. The temperature of the pile where the sample was collected was 57 ºC. Oat husk and pine saw dust were kindly gifted by Avidem (Torrent de Cinca, Huesca, Spain). Oat husk and pine saw dust was mechanically ground to 100 µm. Lignin was purchased from Sigma-Aldrich (Product No. 370959).

### Preparation of substrate flow medium

The nutritional solution of micro-nutrients with interchangeable carbon source described above was used in the enrichment flow medium. Each of the substrates of finely ground oat husk, saw dust, and lignin (4 g/L) was added to batches of nutrient solution. These substrate solutions (containing the soluble fraction of the sample after extraction) were then autoclaved and adjusted to pH 7, and further added to the compost inoculum extract (prepared as described above) to a final concentration of 3% (w/v) in the obtained substrate flow medium.

### Flow experiments

**Fig. 1:**
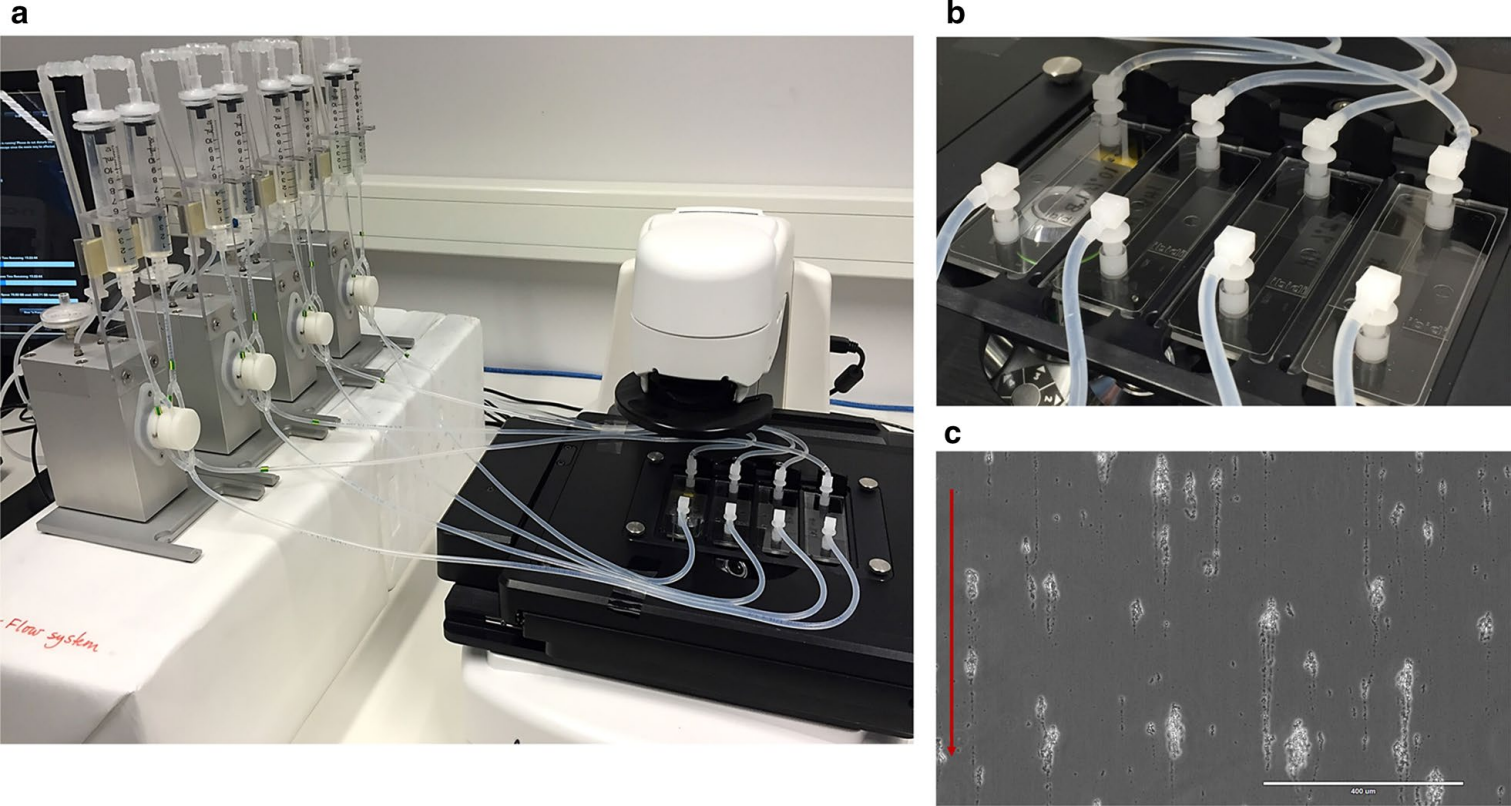
The Flow cell system. Panel **a** Fluidic units with syringes (left) connected to µ-slides mounted in the microscope (right). The syringes are filled with medium that flows continuously over a growth chamber in the µ-slide. Panel **b** Close-up of the four growth chamber µ-slides. Panel **c** Micrograph of biofilm structures with a characteristic tail formed in the growth chamber after 20 days of flow, 10× magnification. The red arrow indicates the flow direction. Example from a lignin-enriched compost biodiversity. Image credit: Gunhild Hageskal

An IBIDI flow cell system (Integrated BioDiagnostics, Martinsried, Germany) was used for the biofilm enrichment studies (Fig. 1). The flow system consists of four fluidic units (holder for medium reservoirs, tubing, and µ-slide) connected to an air pressure pump and is operated by a computer software (IBIDI PumpControl v 1.5.2). One fluidic unit consists of two syringes that are inoculated with a total of 14 ml liquified environmental samples (inoculum flow medium). Each unit has a flow cell slide (µ-slide) with a growth channel (dimensions 50 × 5 mm, 2.5 cm^2^ growth area) where microorganisms can attach to the surface and develop biofilm under a continuous flow of medium. The flow parameters are fully adjustable and can be adapted to a variety of environmental samples. For adaption to soil extract flow medium, the flow parameters were tested and adjusted to a flow rate of 4.2 ml/min and the pressure was set at 10 mbar, which equals a shear stress of 1.46 dyne/cm^2^ in the µ-slide. To ensure optimal biofilm establishment, the µ-slides were connected to an EVOS FL Auto Imaging system (LifeTechnologies™), and real time biofilm development was monitored. Experiments are conducted in a climate laboratory where the whole room is temperature controlled.

### Method demonstration—enrichment experiments

The established method was applied to enrich compost biofilm consortia using three different substrates as carbon source; oat husk, pine saw dust and lignin (final concentration 3% (w/v)). A compost pile sample was used as microbe inoculum, prepared as described for garden soil above, and the biofilm was harvested after 20 days of flow at 30 °C. The flow parameters were adjusted as described in the ‘Flow experiments’ section above, and the microbial consortia of the enriched samples were compared to the original inoculum by sequencing (see below).

### Lysis of biofilm and DNA extraction

Lysis of cells in soil and compost inoculum, and from the shaken-liquid material was done according to Kotlar et al. (2011). Lysis of the biofilm developed in the µ-slide growth channels and DNA isolation was based on the same protocol, modified for use in µ-slides by the following procedure: 200 µl Extraction buffer (EB; 100 mM Tris–HCl (pH8.0), 100 mM sodium EDTA (pH8.0) 100 mM sodium phosphate (pH8.0), 1.5 M NaCl (1% CTAB)) was added to the growth channel of the µ-slide. The slide was sealed with parafilm and sonicated in an ultrasound bath at 37 kHz for 5 min. The sample was then transferred to an Eppendorf tube, added 1.6 µl proteinase K (10 mg/ml), and incubated at 37 °C, 125 rpm, for 30 min. To the growth channel, 200 µl of the EB/proteinase K mixture was added once more to ensure complete harvest of the biofilm cells and proper lysis. The slide was sealed and incubated at 37 °C and 125 rpm for 30 min. Next, 20 µl SDS (20%) was added to both Eppendorf tube and growth channel, followed by an incubation at 60 °C, 125 rpm for 60 min. The lysate was sampled from the growth channel and pooled with the sample in the Eppendorf tube, and then centrifuged at 6000×*g* for 10 min. DNA was extracted from the pooled sample using chloroform:isoamylalcohol (24:1) according to Kotlar et al. (2011). Quantity, quality, and purity of isolated DNA were analysed by Qubit fluorescent measurements (Invitrogen™ Qubit™ dsDNA BR Assay Kit) and microvolume UV–Vis measurements on a DeNovix DS-11 FX + , respectively.

### Barcode sequencing and taxonomic classification

Isolated DNA was subjected to targeted amplicon sequencing of the V3 + V4 region of the 16S ribosomal RNA gene. Sequencing libraries were generated following the Illumina “16S Metagenomic Sequencing Library Preparation guide” (available from: https://support.illumina.com/documents/documentation/chemistry_documentation/16s/16s-metagenomic-library-prep-guide-15044223-b.pdf) with the primers Bakt_341F and Bakt_805R (Herlemann et al. 2011) complemented with Illumina sequencing adapters (5'-TCGTCGGCAGCGTCAGATGTGTATAAGAGACAG-CCTACGGGNGGCWGCAG-3' and 5'-GTCTCGTGGGCTCGGAGATGTGTATAAGAGACAG-GACTACHVGGGTATCTAATCC-3'). The priming part of each primer is underlined, while the Illumina sequencing adapter sequences targeted during barcode indexing by the Nextera XT Index Kit (Illumina) are not. PCR products were purified by Agencourt AMPure XP (Beckman-Coulter) and quantified on a Qubit v2 using the Qubit dsDNA BR Assay Kit (Thermo Fisher Scientific). After sequencing, raw sequencing reads were demultiplexed, filtered, combined, and taxonomically classified by the Metagenomics Workflow within MiSeq Reporter v. 2.5.1 (Illumina), generating abundance tables, which were further processed in Microsoft Excel.

### Shotgun sequencing and CAZy mining

DNA shotgun libraries were generated using the Nextera XT DNA Library Prep kit (Illumina) in combination with the Nextera XT Index Kit (Illumina). Pooled libraries were sequenced on an Illumina MiSeq using the MiSeq Reagent Kit V3 in the 2300 bp paired-end mode. Sequence assemblies were generated in QIAGEN CLC Genomics workbench v.12 with the QIAGEN CLC Microbial Genomics Module plug-in. Raw sequencing reads were quality trimmed by the Trim Reads 2.3 tool using default parameters (Quality limit 0.05, ambiguous limit 2), removing adapter read-through sequences, but keeping broken pairs. The trimmed reads were then assembled by the De Novo Assemble Metagenome 1.0 tool, using default parameters.

The shotgun assemblies were translated in six frames using the *transeq* program of the EMBOSS suite (version 6.6.0.0). The translated sequences were subjected to SINTEF's internal data mining pipeline for mining of carbohydrate active enzymes (CAZymes) using a database of profile Hidden Markov models (HMMs) of CAZymes provided by dbCAN (Yin et al. 2012). The latest version of dbCAN (version 8 at the time of the analysis) can be obtained through web address: http://bcb.unl.edu/dbCAN2/download/Databases/. The mining pipeline is based on *hmmscan* from the software package HMMER version 3.1b2 (http://hmmer.org/) and internally developed programming scripts for post-processing of *hmmscan* results. The mining results were organized in an Access database for data management and query.

## Results

### Method development

**Fig. 2:**
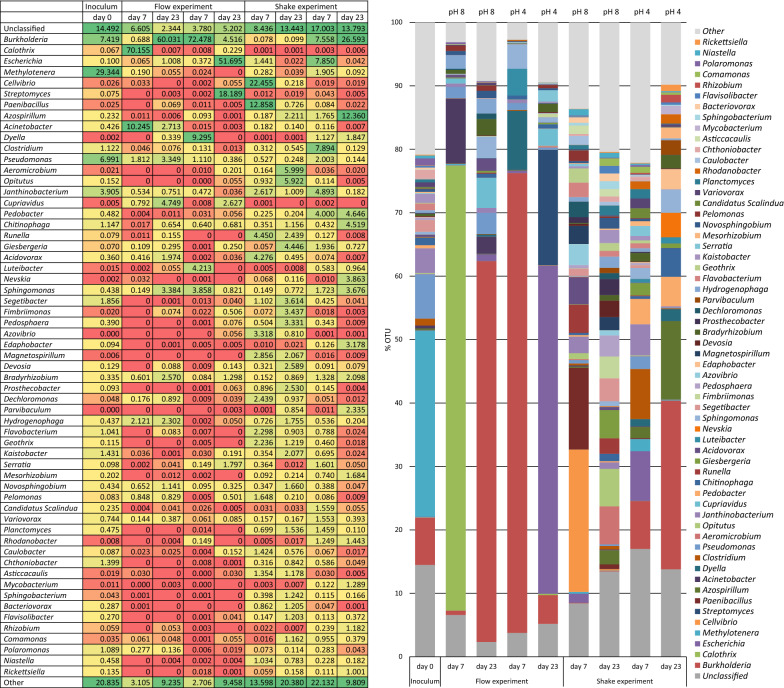
Bacterial consortium composition (genus level) in garden soil samples as analysed by 16S rRNA amplicon sequencing after subjection to different environmental conditions (pH), different biofilm harvest time-points, at 30 °C incubation temperature, and with cellulose as carbon source. Examined with flow and shake experiments and compared to the bacterial composition of the soil inoculum. **a** Heat map presenting the abundancy of dominating genera. **b** Consortium composition of the most dominating genera in inoculum sample as well as after enrichment. “Other” is the sum of all classified genera with individual abundancies below 1% in all samples. “Unclassified” includes sequences classified at higher taxonomic levels, but where no genus information could be assigned

A difference in bacterial consortium composition and abundance, as analysed by 16S amplicon sequencing, was observed in flow experiments compared to shake experiments after enrichment (Fig. 2). The flow-enriched samples were dominated by the genera *Burkholderia*, *Calothrix*, and *Escherichia*, and the abundance of these genera were especially evident compared to the corresponding shake-samples. The enriched consortia were different at day 7 compared to day 23. Especially, there was a shift in abundance of *Burkholderia*, a genus known to include biofilm-forming species. The flow-enriched bacterial consortia were also different at pH 4 compared to pH 8, e.g. at day 23 where taxa belonging to the genus *Escherichia* were most abundant (Fig. 2).

**Fig. 3:**
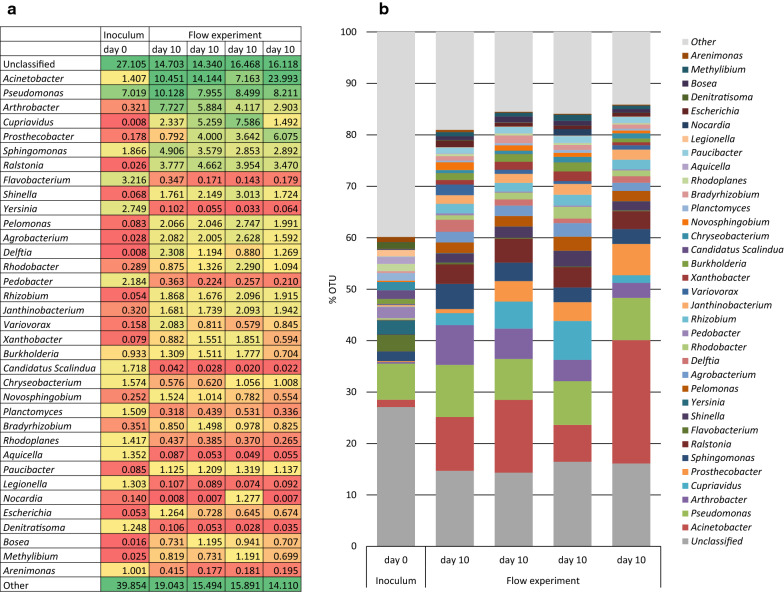
Bacterial consortium composition (genus level) in soil samples analysed by 16S rRNA amplicon sequencing, in four parallel and replicate flow cells with the same environmental conditions (pH 8, 30 °C), same carbon source (cellulose), same inoculum, and same biofilm harvest time-point (after 10 days). **a** Heat map presenting the abundance of dominating genera. **b** Consortium composition of the most dominating genera. “Other” is the sum of all classified genera with individual abundancies below 1% in all samples. “Unclassified” includes sequences classified at higher taxonomic levels, but where no genus information could be assigned

The source of carbon also influenced the consortium composition. Both cellulose (Fig. 2) and glucose (data not shown) were tested at similar environmental conditions, and the results indicated that the bacterial consortium was more diverse with addition of cellulose in the flow medium. Based on 16S rRNA results and real-time imaging, pH 8, 30 °C and 10 days of incubation were identified as good conditions for biofilm development and these were selected for the control experiment with similar environmental conditions and carbon source (cellulose) in four replicate flow cells (Fig. 3). This experiment demonstrated that the consortium compositions in the four replicates were comparable with respect to biofilm microbiota, and that all four samples were dominated by the Genera *Pseudomonas* and *Acinetobacter*, indicating reproducibility of the enrichment experiment as well as low random variation. Please note that the inoculum for the experiment in Fig. 3 is a similar type of sample as the one used for Fig. 2, however not the same/identical sample (as can be seen in the column to the far left in the B section, showing the community in the inoculum).

### Biofilm enrichment of compost with different substrates

**Fig. 4:**
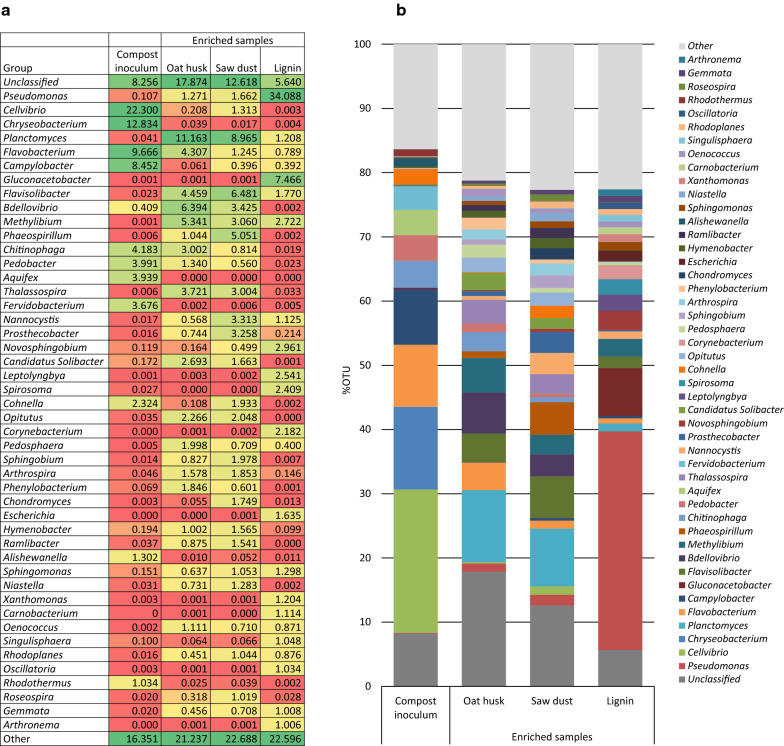
Bacterial consortium composition (genus level) in compost samples as analysed by 16S rRNA amplicon sequencing after enrichment using 3 different substrates (oat husk, pine saw dust, lignin) at 30 °C for 20 days, compared with the compost inoculum. **a** Heat map presenting the abundancy of dominating genera. **b** Consortium composition of the most dominating genera in inoculum sample as well as after enrichment. “Other” is the sum of all classified genera with individual abundancies below 1% in all samples. “Unclassified” includes sequences classified at higher taxonomic levels, but where no genus information could be assigned

The results from the enrichment of compost biodiversity using three different carbon sources clearly demonstrated the applicability of the method, as a large difference in consortium composition was observed in the enriched samples compared to the compost inoculum (Fig. 4). The results also show that enrichment with oat husk and pine saw dust gave similar bacterial consortia compositions after enrichment, whereas use of lignin as carbon source resulted in a domination of bacteria belonging to the genus *Pseudomonas*. In addition, genus *Gluconacetobacter* was exclusively enriched on lignin (Fig. 4A).

### Sequence mining and enzyme hits

**Fig. 5:**
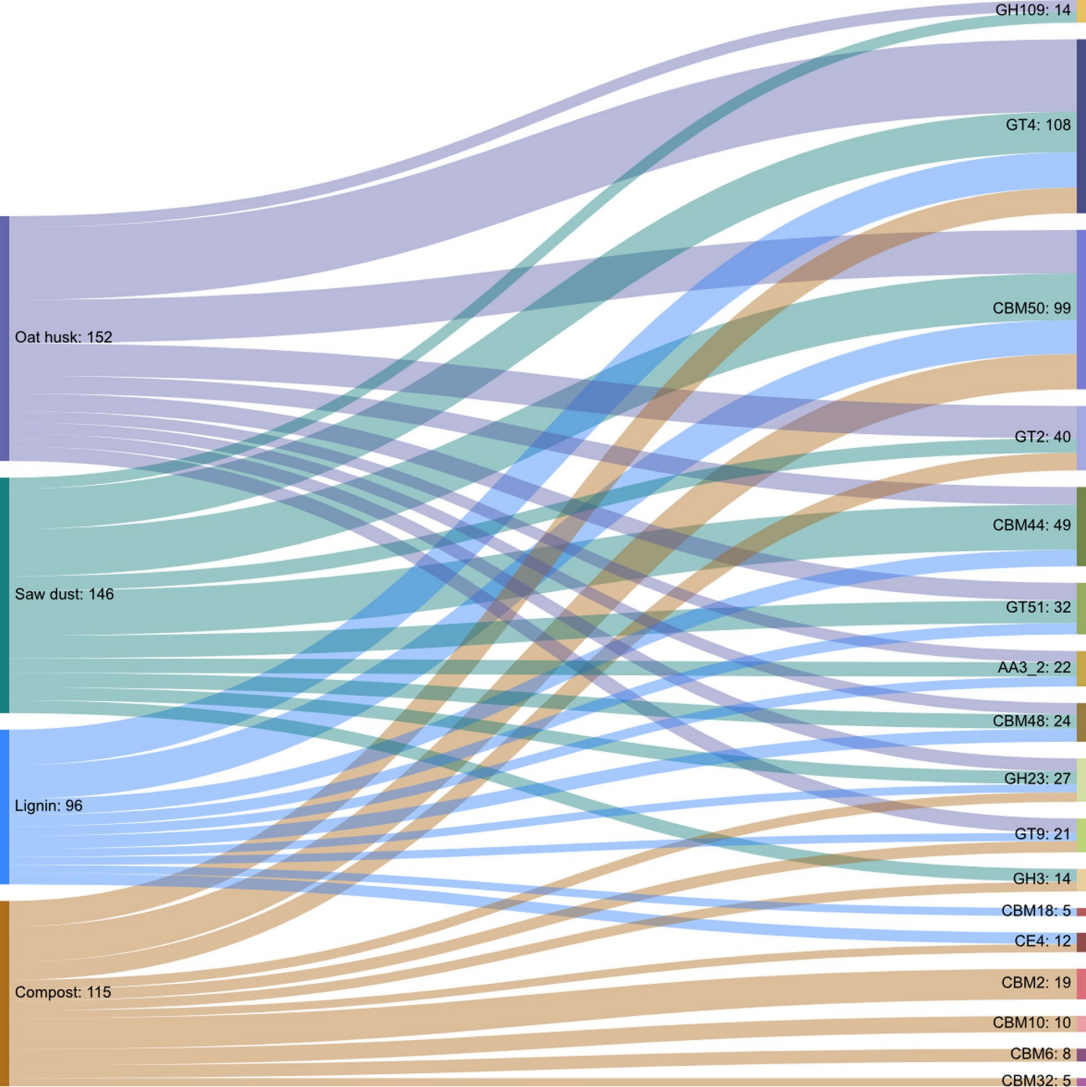
Sankey diagram showing the distribution of hits belonging to the first 10 most abundant CAZyme families in each sample. The total count of hits in each CAZyme family across all the samples and in each sample were indicated after the data labels

Total DNA was isolated from the compost inoculum and the biofilms enriched with oat husk, pine saw dust, and lignin substrates, and analysed by shotgun sequencing. The metagenome sequence assemblies generated were then mined for CAZymes, and a total of 1127 hits across different CAZyme families were identified. Analysis revealed differences in hits of CAZyme families among the enriched samples, as well as differences between the original compost inoculum and the enriched samples. The distribution of total hits of the ten most abundant CAZyme families in all samples is described in detail in Table 1, as well as shown in Fig. 5.

**Table 1:** Detailed breakdown of the 10 most abundant CAZyme hits belonging to the different enriched samples and compared to the original compost inoculum. Results presented as number of CAZyme family hits per sample

CAZyme family		Count per CAZyme family in samples			
		Compost inoculum	Oat husk	Saw dust	Lignin
Glycosyltransferase	GT2	11	20	9	–
	GT4	16	45	25	22
	GT9	7	9	–	5
	GT51	–	11	14	7
Glycoside hydrolase	GH3	6	–	8	–
	GH23	6	8	8	5
	GH109	–	7	7	–
Carbohydrate-binding module	CBM2	19	–	–	–
	CBM6	8	–	–	–
	CMB10	10	–	–	–
	CBM18	–	–	–	5
	CBM44	–	11	28	10
	CBM48	–	7	9	8
	CBM50	22	27	29	21
Carbohydrate esterase	CE4	5	–	–	7
Auxiliary activity	AA3_2	–	7	9	6
	Total No. of family hits	110	152	146	96

Among the CAZymes that were identified using the data mining pipeline, the most abundant families in each of the samples were glycoside hydrolases (GH), glycosyltransferases (GT), and carbohydrate-binding modules (CBM) (Fig. 5). Other families, e.g. carbohydrate esterase (CE) and auxiliary activity family 3 (AA3) were less abundant, while CE4 and AA3_2 were exclusively found in the compost inoculum and in the substrate enriched samples, respectively (Table 1). Compared to the original compost inoculum, oat husk and pine saw dust enriched samples also differed in the GH families, and some GT families, such as GT51 (murein polymerase activity; http://www.cazy.org/GT51.html), were only found in substrate enriched samples. Oat husk and pine saw dust enriched samples had the largest number of CAZyme family hits, except for the CBM family in which the compost inoculum dominated in both numbers and diversity, although CBM44 (cellulose- and xyloglucan-binding function; http://www.cazy.org/CBM44.html) and CBM48 (glycogen-binding function; http://www.cazy.org/CBM48.html) were only found in the enriched samples. The lignin enriched sample had less diverse CAZyme family hits compared to saw dust and oat husk enriched samples, as well as a lower number of total identified hits (Table 1). Figures of detailed distribution of enzyme hits in the GH, GT, CBM, and CE families identified in the samples are presented in the additional file.

## Discussion

A flow-based method for enrichment of biofilm communities was successfully established and subsequently used to enrich metagenomes from a hot compost pile to demonstrate its function. The method can be adapted to other relevant samples, environmental conditions, and nutrient preferences of choice, targeting enzymes or enzyme classes of interest. The results from the 16S rRNA sequencing show a difference in consortium composition in flow experiments compared to shake experiments after enrichment. The most pronounced difference was seen with the shift in relative abundances in the microbial consortia analysed, reducing complexity and increasing relative abundance of some taxa in flow-experiments, indicating that the flow-based method are effective in enriching biofilm organisms, which fulfil the goal to approach natural conditions for increased biofilm development. The flow-based method is beneficial for reducing bacterial consortia complexity by selecting biofilm-forming bacteria, as most of the enriched taxa (i.e. taxa increased in abundance after flow experiment) are known to be biofilm-producing (e.g. *Burkholderia*). Hence, it is possible to enrich the biofilm community from a sample, and also to enrich it in various directions based on the choice of sample material, environmental conditions, and nutritional preferences, exemplified by enriching the microbes able to metabolise cellulose by using cellulose as the carbon source in the flow experiment. The system can therefore be used to reduce the complexity of diverse samples, increasing the fraction of the established microbiome by selecting for taxa able to form biofilm, as well as use a certain carbon-source or cope with certain environmental conditions, such as pH, salt concentration or temperature, dependent on the properties desired.

Lignin is rather resistant towards degradation, which may explain the difference in bacterial consortium composition in the lignin enriched sample compared to the oat husk and pine saw dust enriched samples, containing supposedly more accessible carbon sources like cellulose and hemicellulose, that can be utilized by a wider range of bacteria. This was also reflected in the results from the enzyme gene sequence mining, where the lignin enriched samples were less diverse in CAZyme families and had relatively few identified hits. Both oat husk and saw dust are rich in carbon that is probably more available and easily degradable for bacteria and stimulates growth, hence resulting in more diverse microbial metabolism. The dominating group of *Pseudomonas* spp. that were observed in the lignin enriched samples may merely be better adapted to nutrient deficiency as a function of the biofilm community, or they may also be able to produce enzymes capable of lignin modification, which has been reported in this genus previously (Bugg et al. 2011; de Gonzalo et al. 2016; Ravi et al. 2019; Sun et al. 2018). The lignin-enriched *Gluconacetobacter* spp. have been previously reported to produce cellulose, and to contribute to polymerization of lignin (Touzel et al. 2003), and may also harbour enzymes capable of lignin depolymerization. In the light of the high demand for efficient and cost-effective methods for selective lignin degradation, these are interesting results and demonstrates the applicability of the enrichment method.

The sequence mining results demonstrated that enzyme classes such as different GHs, can be targeted by using biofilm enrichment of a microbial community with different substrates. Some CAZyme families, such as GTs, were only found in substrate enriched samples, which demonstrated the potential of obtaining diverse or targeted enzyme classes by using the developed method. The differences observed in CAZyme family types both between different substrate-enriched samples, and also between the compost inoculum and the enriched samples, show that the described method can be used to enrich microorganisms and subsequently the types of enzymes that are active on certain types of substrates. As an example, the developed method was demonstrated by successfully enriching CBM44, CBM48 and GT51 from the compost biodiversity using relevant substrates. CBM44 binds with equal affinity to cellulose and xyloglucan (Najmudin et al. 2006) and was enriched using all substrates, but particularly when using pine saw dust. This fits well with the assumption of the presence of cellulose compounds in all enrichment substrates compared to the natural habitat, and in particular in saw dust. CBM48, a family associated with binding of starch (Holck et al. 2019), was also enriched using all substrates applied, however to a lower degree compared to CBM44. Various CBM families identified in the samples were previously known to associate with enzymes from GH families (Boraston et al. 2004; Janeček et al. 2019; Shoseyov et al. 2006) and the abundance of CBM families is well correlated with the diversity of GH families observed. Members in the AA3 CAZyme family were found exclusively in enriched samples and they are diverse enzymes catalysing the oxidation of alcohols or carbohydrates. These enzymes are abundantly found in wood degrading fungi, typically involved in lignocellulosic degradation (Sützl et al. 2018), aligning well with the observed enrichment of such enzymes in these enrichment media.

As previous reported by Wang et al. (2016), enzymes from the GT families are abundant in microbial communities enriched from compost. The enzymes identified from the enriched samples mainly involve in the synthesis of mono-, oligo- (GT4), and polysaccharides (GT2), as well as cell membrane synthesis (GT51), supporting the hypothesis that these enzymes can play a role in biofilm formation during enrichment. Some modelled 3D structures from GT51 are lysozyme-like, indicating a potential role in biofilm formation (Lairson et al. 2008; Yuan et al. 2007). A notable divergence of CAZymes could be observed in the GH families. Many GH families, known to have cellulase or hemicellulases activity, were found exclusively in compost inoculum, whereas some GH families having oligosaccharide degrading activity were identified only in oat husk and/or pine saw dust enriched samples. These observations indicate that in complex and partially degrading cellulosic materials like compost, cellulases or hemicellulases will be more favourable as they are active in further degradation of cellulose and hemicellulose, whereas in oat husk and saw dust (being finely ground powder), enzymes degrading smaller polysaccharide fractions might be enriched. GH enzymes were as expected extensively enriched using lignin substrate, and are likely to be enriched due to their activity on cellulosic residues still present in the sample. For further details and figures of the distribution of the discussed enzymes classes within the enriched samples, please refer to the Supplementary file.

With the described method, applying biofilm-based enrichment of environmental samples at different conditions, one will be able to target interesting genes and enzymes as candidates for use in various processes, such as decomposition of different waste materials, thus biofilm enrichment could aid the discovery of e.g. hydrocarbon degraders and exopolysaccharide producers by increasing the density of microbes harbouring these functions from environmental samples subjected to targeted enrichment. In the long term, enrichment methods such as this, combined with metagenome technologies may help initiate innovations in environment, agriculture, energy, health, and in industry applications.

## Data Availability

The datasets generated and analysed during the current study are available from the corresponding author on reasonable request, and raw sequencing reads have been submitted to the NCBI Sequence Read Archive (SRA) and are available in Bioproject PRJNA742182 (http://www.ncbi.nlm.nih.gov/bioproject/742182). Some results data are also included as additional file.
